# Evaluation of the IMMULITE^® ^2000 CMV IgM assay

**DOI:** 10.1186/2042-4280-3-2

**Published:** 2012-02-29

**Authors:** Tricia A Bal, Glenn Armstrong, Xiang Y Han

**Affiliations:** 1 Siemens Healthcare Diagnostics, Los Angeles, CA, USA; 2 University of Texas MD Anderson Cancer Center, Houston, TX, USA

## Abstract

**Background:**

Diagnosis of cytomegalovirus (CMV) infection is challenging because of the high rate of asymptomatic infection and the low specificity of associated symptoms and signs. As a result, laboratory testing is an essential aid in making an accurate diagnosis. The presence of CMV IgM is indicative of primary CMV infection. In pregnancy, diagnosis of primary infection is important because primary maternal infection increases fetal infection risk substantially. Fetal infection can result in serious sequelae ranging from neurological deficits to death. Diagnosis among the immunocompromised is also critical for the timely initiation of therapy that can reduce morbidity and mortality risk.

**Methods:**

The IMMULITE^® ^2000 CMV IgM assay qualitatively detects CMV IgM antibodies in human serum or plasma to aid in the diagnosis of current or recent CMV infection. To determine expected values in apparently healthy subjects, 136 samples were tested. Reproducibility, normal range, and method comparison studies were also performed to evaluate the assay's performance. The assay's reproducibility was evaluated across three sites. Seven hundred and eighteen (n = 718) individual patient serum samples, which included samples from CMV IgM-positive (n = 109, determined by the Abbott IMx CMV or the Diamedix CMV IgM assays), pregnant (n = 210), HIV-positive (n = 30), immunosuppressed (n = 102), and transplant patients (n = 17) and from patients with potentially cross-reacting conditions (n = 136) were evaluated in the method comparison study. The positive, negative, and overall agreement between the IMMULITE 2000 CMV IgM assay and the VIDAS CMV IgM assay (predicate assay) were determined.

**Results:**

The assay demonstrated excellent reproducibility with a total CV of less than 10%. The positive, negative, and overall agreement between the IMMULITE 2000 assay and the VIDAS assay were > 95% for the method comparison samples. Among potentially cross-reactive samples, the overall agreement between the two assays was 96%. Similarly, among the immunocompromised and pregnant subjects, the overall agreement was ~96% and ~97%, respectively.

**Conclusions:**

The IMMULITE 2000 CMV IgM assay demonstrated excellent reproducibility, minimal cross-reactivity, and performance comparable to that of the VIDAS CMV IgM assay. It can aid in the diagnosis of acute CMV or recent CMV infection by qualitatively detecting the CMV IgM antibodies in human serum or plasma.

## Background

Cytomegalovirus (CMV) infection affects all age groups, and there is no vaccine available. It is spread through direct exposure to infected body fluids such as urine, blood, breast milk, and saliva or via transplant of infected organs [1,2]. CMV infections in healthy individuals are generally asymptomatic, and when symptoms occur they are typically mild, nonspecific and self-limiting. As a result, diagnosis of CMV infection is challenging, and laboratory testing is an essential aid in making an accurate diagnosis.

In pregnancy and among the immunocompromised, CMV infection can have severe consequences, and thus, in these groups, laboratory testing is a vital aid in facilitating timely, appropriate interventions. Cytomegalovirus is the leading cause of congenital viral infection worldwide, with an incidence of 0.5% to 3% of live births [3,4]. It is also a leading cause of deafness and mental retardation. Transmission rates from mother to fetus are dependent on the type of maternal infection. In pregnancies with primary infection, the rate of fetal infection is approximately 32% to 38% [1]. In comparison, in pregnancies with reactivation or reinfection, the rate of fetal infection is only ~1% [1-3].

Infection in early pregnancy is associated with more severe sequelae, and congenital CMV disease is most likely after a primary maternal infection [1,3,4].

Among the immunocompromised, CMV infection (primary, reinfection, or reactivation) is associated with increased morbidity and mortality. Symptomatic CMV infection occurs in 20% to 60% of transplant recipients [5].

Diagnosis of CMV infection and the type of infection is based on a combination of tests, including tests that measure or detect IgM, IgG, or IgG avidity. CMV IgM plays an important role in the diagnosis of CMV infection. The presence of IgM is indicative of acute or primary infection, and the presence of IgG is indicative of past infection. When both IgM and IgG are positive, the level of IgG avidity is used to distinguish acute/primary infection from past infection [4]. This study assessed the performance of the fully automated IMMULITE 2000 CMV IgM assay.

## Methods

The IMMULITE 2000 CMV IgM assay is for the qualitative detection of IgM antibodies to CMV in human serum or plasma, as an aid in the diagnosis of current or recent CMV infection (Table 1). It is an automated solid phase enzyme-linked chemiluminescent three-step immunoassay. The VIDAS^® ^CMV IgM assay is intended to be used as an aid in the diagnosis of cytomegalovirus infection (Table 1). It is an automated enzyme-linked fluorescent immunoassay for the qualitative detection of CMV IgM antibodies in human serum. Both the IMMULITE and VIDAS CMV IgM assays were performed according to the manufacturer's instructions.

**Table 1:** Cutoffs of the VIDAS and IMMULITE 2000 CMV IgM assays

VIDAS		IMMULITE 2000	
**Signal/Cutoff Ratio**	**Interpretation**	**Signal/Cutoff Ratio**	**Interpretation**

< 0.7	Negative	< 0.90	Nonreactive

≥ 0.7 to < 0.90	Equivocal	0.9 to < 1.1	Indeterminate

≥ 0.90	Positive	≥ 1.1	Reactive

To evaluate the IMMULITE 2000 CMV IgM assay's performance, reproducibility, normal range, and method comparison studies were performed.

The reproducibility study was conducted at three sites using serum pools. Three serum pools were used: high negative (70%-80% of the cutoff), around the cutoff (110%-120% of the cutoff), and medium/high positive (200%-250% of the cutoff). Two runs per day were conducted over 5 days at each site. Each run included four replicates of each serum pool for a total of 40 replicates per sample at each site. Negative and positive kit controls were included in each run for quality control.

To determine expected values in apparently healthy subjects, 136 serum specimens were collected from healthy asymptomatic subjects with no known evidence of exposure to CMV. Samples were collected with informed consent and Institutional Review Board approval.

The method comparison study compared the IMMULITE 2000 CMV IgM assay to the VIDAS CMV IgM assay. A total of 718 individual patient serum samples were tested. These included samples from from CMV IgM-positive (109 determined by the Abbott IMx CMV IgM or the Diamedix CMV IgM assays), pregnant (210), HIV-positive (30), immunosuppressed (102), and transplant patients (17), and from patients with potentially cross-reactive conditions (136). Potentially cross-reacting conditions were determined based on the results of FDA-cleared immunoassays and included 60 antinuclear antibody (ANA), 23 Epstein-Barr virus (EBV), 3 herpes simplex, 25 rheumatoid factor (RF), and 25 rubella positive samples. In the analysis of sample subgroups, some samples qualified for multiple subgroup classifications. Positive, negative and overall agreement and 95% confidence intervals were determined. Equivocal (VIDAS) and indeterminate (IMMULITE) results were excluded from the agreement analysis.

## Results

The IMMULITE 2000 CMV IgM assay demonstrated excellent reproducibility, minimal cross-reactivity, and performance comparable to that of the predicate assay. The 95th percentile values in apparently healthy subjects supported the validity of the assay's cutoffs.

### Reproducibility

The IMMULITE 2000 assay demonstrated a total CV across three sites of less than 10% (Table 2).

**Table 2:** Reproducibility determined over 5 days at three sites using 120 replicates per pool

Sample	Days (n)	Mean (signal/cutoff)	Within-Run (%)	Between-Run (%)	Between-Site (%)	Total (%)
Pool 1	5	0.63	4.3	2.4	7	8.6

Pool 2	5	1.06	5.4	5.2	0.3	7.5

Pool 3	5	2.09	5.4	5.6	2.9	8.3

### Reference range among the apparently healthy

Among healthy subjects, the 95th percentile signal/cutoff value of 0.66 was supportive of the assay's negative cutoff of 0.9 (Table 3).

**Table 3:** The mean, median, and 95th percentile values in apparently healthy subjects by gender

Gender	n	95th Percentile	Mean (95% CI)	Median (95% CI)
Female	62	0.77	0.27 (0.21 to 0.33)	0.19 (0.15 to 0.30)

Male	74	0.66	0.22 (0.17 to 0.26)	0.16 (0.13 to 0.18)

Total	136	0.66	0.24 (0.21 to 0.27)	0.17 (0.14 to 0.19)

### Method comparison

The positive, negative, and overall agreement between the IMMULITE 2000 assay and the VIDAS assay for the method comparison study population (n = 718) was > 95% (Table 4).

**Table 4:** Overall method comparison results for the IMMULITE 2000 and VIDAS CMV IgM assays

	VIDAS			
**IMMULITE 2000**	**Positive**	**Equivocal**	**Negative**	**Total**

**Reactive**	98 (13.6%)	7 (1.0%)	9 (1.3%)	114 (15.9%)

**Indeterminate**	1 (0.1%)	2 (0.3%)	7 (1.0%)	10 (1.4%)

**Nonreactive**	3 (0.4%)	9 (1.3%)	582 (81.1%)	594 (82.7%)

**Total**	102 (14.2%)	18 (2.5%)	598 (83.3%)	718 (100%)

**Agreement**	**IMMULITE / VIDAS**		**95% CI**	

**Positive**	98/102 (96.1%)		90.3 to 98.9	

**Negative**	582/598 (97.3%)		95.7 to 98.5	

**Overall**	682/718 (95.0%)		93.1 to 96.5	

### Potentially cross-reactive samples

In patients with potentially cross-reacting samples (n = 136), the IMMULITE 2000 CMV IgM assay had one reactive sample and two indeterminate samples; similarly, the VIDAS CMV IgM assay had one positive sample and two equivocal samples. The samples that were VIDAS CMV IgM positive and equivocal were ANA positive. One IMMULITE CMV IgM reactive sample was RF positive. Of the IMMULITE CMV IgM indeterminate samples, one was ANA positive and the other was RF positive. The overall agreement between the two assays was approximately 96% (Table 5).

**Table 5:** IMMULITE 2000 and VIDAS assays results and agreement from patients with potentially cross-reactive samples

Agreement	IMMULITE / VIDAS	95% CI
**Positive**	0/1 (0%)	0 to 97.5

**Negative**	131/133 (98%)	94.7 to 99.8

**Overall**	133/136 (96%)	91.6 to 98.8

### Immunocompromised patients

Overall agreement between the IMMULITE 2000 assay and the predicate assay was ~96%, for the immunosuppressed, ~97% for the HIV-positive, and ~92% for transplant subjects (Tables 6, 7, and 8). In the immunosuppressed population, the VIDAS assay had one positive and one equivocal result and the IMMULITE assay had two indeterminate results. In the HIV-positive subjects, the VIDAS assay had one positive and one equivocal result and the IMMULITE assay had one reactive result. Among transplant subjects, the VIDAS assay had one equivocal result.

**Table 6:** IMMULITE 2000 and VIDAS CMV assay results and agreement for samples from immunocompromised subjects

Agreement	IMMULITE / VIDAS	95% CI
**Positive**	0/1 (0.0%)	0 to 97.5

**Negative**	87/89 (97.8%)	92.1 to 99.7

**Overall**	87/91 (95.6%)	89.1 to 98.8

**Table 7:** IMMULITE 2000 and VIDAS CMV assay results and agreement for samples from HIV-positive subjects

Agreement	IMMULITE / VIDAS	95% CI
**Positive**	1/1 (100.0%)	2.5 to 100

**Negative**	27/27 (100.0%)	87.2 to 100

**Overall**	28/29 (96.6%)	82.2 to 99.9

**Table 8:** IMMULITE 2000 and VIDAS CMV assay results and agreement for samples from transplant subjects

Agreement	IMMULITE / VIDAS	95% CI
**Positive**	0/0 (Undefined)	Not applicable

**Negative**	11/11 (100.0%)	71.5 to 100

**Overall**	11/12 (91.7%)	61.5 to 99.8

### Pregnancy

Overall agreement between the IMMULITE 2000 assay and the predicate assay was approximately 97% (Table 9). The VIDAS assay had two reactive and two equivocal results and the IMMULITE assay had three reactive and three indeterminate results.

**Table 9:** IMMULITE 2000 and VIDAS CMV assay results and agreement for samples from pregnant subjects

Agreement	IMMULITE / VIDAS	95% CI
**Positive**	1/2 (50.0%)	1.3 to 98.7

**Negative**	175/179 (97.8%)	94.4 to 99.4

**Overall**	177/183 (96.7%)	93.0 to 98.8

## Discussion

CMV IgM can be detectable during new infection, reactivated infection, or infected individuals who have been recently re-exposed [4,6]. CMV IgM can aid in the diagnosis of primary infection, and it should be used in conjunction with other clinical information.

The IMMULITE 2000 CMV IgM assay demonstrated good reproducibility around the assay cutoffs with maximum total CV of 8.6% providing precise results. The 95^th ^percentile value of 0.66 in apparently healthy subjects was far below the negative cutoff value of 0.90 supporting this cutoff level. The IMMULITE CMV IgM assay demonstrated comparable performance with the VIDAS CMV IgM assay with an overall agreement in the method comparison study population (n = 718) of 95%. Among the various subpopulations studied, the overall agreement ranged from 91.7% to 96.7%.

Among patients with cross-reacting conditions, interpretation of CMV IgM results may be challenging because of the potential for false-positive results. In this study of the 136 samples from patients with potentially cross-reacting conditions, the reactive/positive and indeterminate/equivocal results were from patients who were either ANA or RF positive (IMMULITE 2000 CMV IgM assay 3 results and VIDAS CMV IgM 3 results).

CMV infection can be serious and even fatal in pregnancy and in the immunocompromised [3-9]. Diagnosis of active CMV infection can be accomplished through detection of CMV DNA in body fluids; this approach plays an important role in the immunocompromised [8,9], and infants at risk for congenital disease [4]. This study demonstrated a 92% to 96% agreement between the IMMULITE 2000 and VIDAS CMV IgM assays among immunocompromised patients. Among immunocompromised (transplant, HIV-infected or immunosuppressed) individuals CMV IgM and/or IgG antibodies may be difficult to detect because of low titers, thus a negative serology result should not be used in isolation to rule-out infection [8].

CMV IgG seroprevalence, indicative of past infection, among women of childbearing age ranges from 50% to > 80% [4]. While seroconversion using only IgG antibodies can be used to help determine when primary infection occurred, this is typically done retrospectively, whereas, CMV IgM testing may aid in the diagnosis of primary infection concurrently.

Diagnosis of primary CMV infection in pregnancy is vital for establishing fetal infection risk because fetal infection risk and serious sequelae are higher for primary infections [3,4]. Women who are seronegative for CMV are at risk for contracting a primary infection during pregnancy and have a much higher risk of transmitting the virus to the fetus or neonate [3,4]. Furthermore, transmission of the virus early in pregnancy is associated with worse fetal outcomes [3]; thus CMV IgM testing may facilitate earlier identification of high-risk pregnancies and earlier fetal testing. The IMMULITE 2000 CMV IgM assay demonstrated excellent overall agreement, 96.6%, with the VIDAS CMV IgM assay for identification of primary infection in pregnant subjects.

## Conclusions

The IMMULITE 2000 CMV IgM assay demonstrated excellent reproducibility, minimal cross-reactivity, and performance comparable to that of the VIDAS CMV IgM assay. The sample population included pregnant women; immunosuppressed, transplant, and HIV-positive patients; and individuals with potentially cross-reactive infections. The IMMULITE 2000 CMV IgM assay is a tool that can aid clinicians in the diagnosis of CMV infection. When it is combined with the IMMULITE 2000 CMV IgG assay and other IMMULITE 2000 ToRCH tests; it can help laboratories optimize consolidation of their ToRCH testing.

## Competing interests

The authors declare that they have no competing interests.

## Authors' contributions

GA compiled the initial study report and provided critical editorial comments. TAB reviewed the data and created the manuscript. XYH provided critical editorial comments and performed some of the studies. All authors read and approved the final manuscript.
